# Case Report: Sympathetic nerve block treat chronic kidney disease-associated pruritus

**DOI:** 10.3389/fnins.2025.1529183

**Published:** 2025-04-16

**Authors:** Ying Li, Yaohua Chen, Jingyi Nie, Xiaoguang Huang, Weiwei Zhang

**Affiliations:** ^1^Department of Anesthesiology, Third Hospital of Shanxi Medical University, Shanxi Bethune Hospital, Shanxi Academy of Medical Sciences, Tongji Shanxi Hospital, Taiyuan, China; ^2^Department of Anesthesiology, Shanxi Medical University, Taiyuan, China; ^3^Department of Nephrology, Third Hospital of Shanxi Medical University, Shanxi Bethune Hospital, Shanxi Academy of Medical Sciences, Tongji Shanxi Hospital, Taiyuan, China

**Keywords:** CKD-aP, uremia, sympathetic nerve block, chronic kidney disease, autonomic nervous system

## Abstract

Uremia is the advanced stage of chronic kidney disease (CKD), characterized by the accumulation of metabolic waste products in the blood as a result of the kidneys' inability to meet the body's metabolic demands. Patients with uremia frequently experience a range of complications, including renal anemia, osteoporosis, and cardiovascular disease, all of which significantly impair their quality of life. Chronic kidney disease-associated pruritus (CKD-aP) is a common comorbidity in uremic patients, particularly among those undergoing hemodialysis, with prevalence rates ranging from 10 to 77%. This case report enrolled three patients diagnosed with chronic kidney disease-associated pruritus (CKD-ap) who underwent ultrasound-guided sympathetic nerve blockade procedures. The results demonstrated significant reductions in pruritus intensity concurrent with measurable enhancements in sleep quality and daily functioning.

## 1 Introduction

Uremia is the advanced stage of chronic kidney disease (CKD), characterized by the accumulation of metabolic waste products in the blood as a result of the kidneys' inability to meet the body's metabolic demands (Levey et al., 2003). Patients with uremia frequently experience a range of complications, including renal anemia, osteoporosis, and cardiovascular disease, all of which significantly impair their quality of life (Joseph et al., 2020; Sulková et al., 2021; Coronado Daza et al., 2015). Chronic kidney disease-associated pruritus (CKD-aP) is a common comorbidity in uremic patients, particularly among those undergoing hemodialysis, with prevalence rates ranging from 10 to 77% (Makar et al., 2021). Research has indicated that the deposition of metabolic waste products, neurological dysfunction, microinflammation, and immune system imbalance are closely associated with the development of CKD-aP (Kimmel et al., 2006; Agarwal et al., 2021).

The role of sympathetic nerves has garnered significant attention in the investigation of the pathogenesis of CKD-aP. Sympathetic nerves are a critical component of the autonomic nervous system and are involved in the regulation of both acute and chronic pain through mechanisms such as neurological and endocrine interactions, as well as modulation of nutrition and immunity (Doroshenko et al., 2024). It has been proposed that abnormal sympathetic nerve activity is closely linked to pruritus, particularly in the context of neuropathic itch, with dysregulation of this activity potentially contributing to the intensification of itch, thereby exacerbating cutaneous symptoms (Tong et al., 2022).

For CKD-aP, optimal treatment remains unclear (Simonsen et al., 2017). Clinically, gabapentin and naltrexone are the most commonly prescribed medications for alleviating CKD-aP. However, these drugs frequently cause adverse effects such as dizziness, leading to poor patient compliance. Alternative therapies, including phototherapy and acupuncture, often require prolonged treatment durations and exhibit slow onset of efficacy, which can impose significant financial burdens on patients (Siemens et al., 2014; Mettang and Kremer, 2015). Clinicians typically utilize either single or combination therapies, considering the severity of symptoms to achieve the most effective therapeutic outcomes. Sympathetic nerve block, a well-established treatment, has been proven safe and effective in managing conditions such as complex regional pain syndrome, post-traumatic stress disorder, and cardiac arrhythmias (O'Connell et al., 2016; Lipov and Ritchie, 2015; Witt et al., 2017). We hypothesized that sympathetic blockade might alleviate pruritus by suppressing abnormal sympathetic nerve activity in CKD-aP patients, thereby improving their quality of life. To test this hypothesis, we recruited three uremic patients suffering from pruritus, with the expectation that their responses could provide new insights into the treatment of CKD-aP.

## 2 Case report

### 2.1 Case 1

The patient was a 70-year-old female with chronic renal failure in the uremic stage, complicated by grade 2 hypertension, type 2 diabetes mellitus, severe anemia, and several other underlying conditions. She was admitted to our hospital for hemodialysis due to progressively elevated serum creatinine, electrolyte disturbances, and acid-base imbalances. During hemodialysis, she experienced episodes of nosebleeds, as well as adverse reactions including nausea, vomiting, and insomnia. Prior to treatment, the patient suffered from persistent pruritus, particularly at night, affecting multiple areas such as the face, trunk, and limbs. Additionally, she often experienced difficulty falling asleep, sleep disturbances, and poor blood pressure control, which required increased doses of antihypertensive medication. When the pruritus symptoms became severe, the patient was administered gabapentin orally for symptomatic management. However, the therapeutic response was unsatisfactory. Prior to undergoing sympathetic nerve block therapy, the patient had discontinued the medication for 2 weeks.

### 2.2 Case 2

The patient, a 61-year-old male, was in the uremic stage of chronic renal failure, complicated by grade 3 hypertension, type II diabetes mellitus, diabetic nephropathy, renal anemia, renal bone disease, and multiple other comorbidities. In recent years, he had experienced persistent pruritus, generalized fatigue, recurrent bilateral lower extremity edema, intermittent morning nausea, and other symptoms, all of which were accompanied by progressively elevated blood creatinine, hyperkalemia, and repeated peritoneal dialysis treatments. Recent refinement of the peritoneal balance test indicated inadequate dialysis, leading to adjustments in the patient's peritoneal dialysis regimen. Subsequently, venous fistuloplasty was performed, and it was recommended that the patient transition to hemodialysis once the arteriovenous fistula matured. Prior to treatment, the patient exhibited significant pruritus on his back and face, with visible signs of scratching upon examination. The symptoms were exacerbated during dialysis and significantly aggravated by cold, heat, and increased stress. No corresponding symptomatic treatment was administered to the patient for the pruritus symptoms.

### 2.3 Case 3

The patient is a 70-year-old female who, in 2017, developed generalized edema without an obvious causative factor. Laboratory tests revealed elevated blood creatinine levels, leading to a diagnosis of chronic renal failure. She has since been undergoing regular dialysis treatment and has progressed to the uremic stage. The patient also has a comorbid diagnosis of type II diabetes mellitus. Prior to treatment, she experienced significant pruritus in the limbs and back, which was initially alleviated by hydration agents but became progressively less effective over time. The persistent pruritus severely impacted her sleep quality, leading to mood swings and irritability, which significantly compromised her overall quality of life.

## 3 Results and discussions

**Figure 1:**
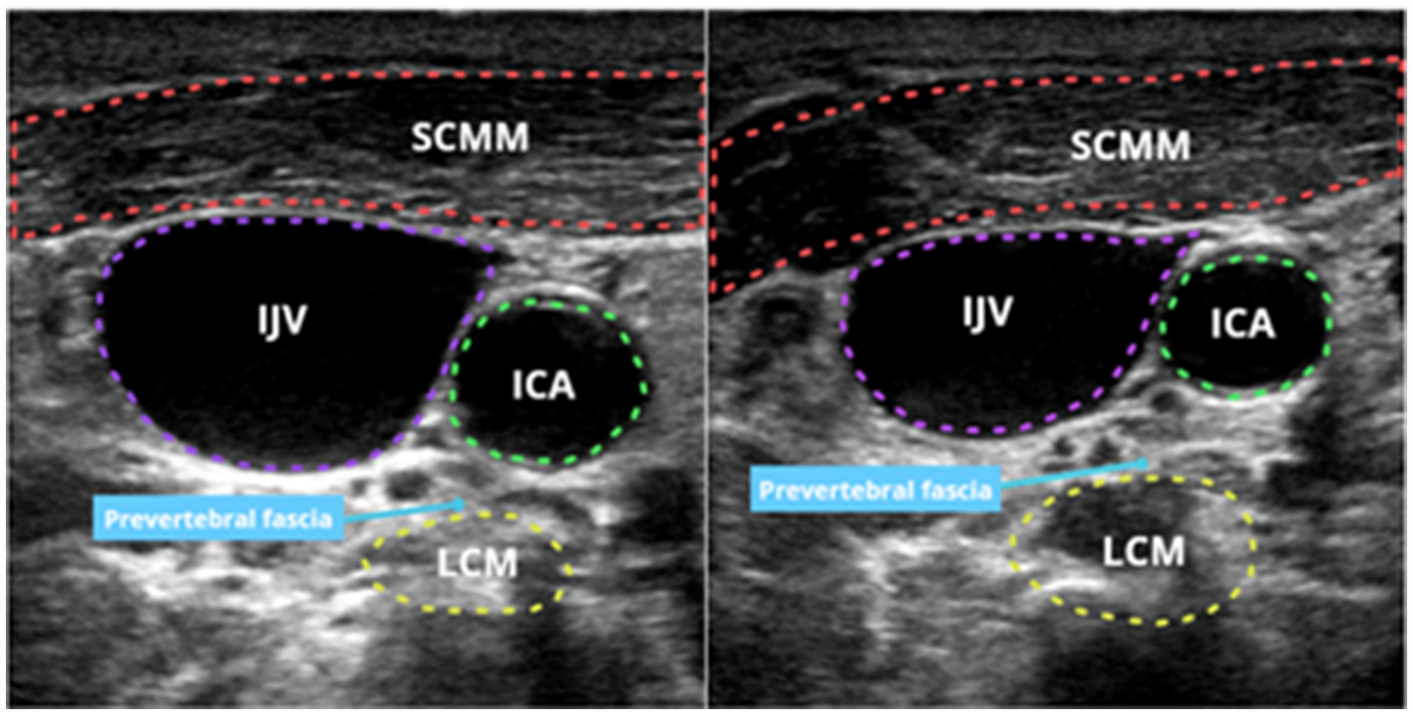
Ultrasound-guided stellate ganglion block before and after.

All three patients presented with severe pruritus, which was not observed prior to the diagnosis of CKD. The intensity of their pruritic symptoms worsened with the progression of renal disease, prompting a diagnosis of CKD-aP (Verduzco and Shirazian, 2020). Following full informed consent, we performed a sympathetic nerve block under the guidance of one of our experienced anesthesiologists. The procedure was conducted as follows (Figure 1): under ultrasound guidance, a needle was inserted near the 6th cervical vertebra, targeting the anterior ramus of the transverse process at a depth of 3.0–3.5 cm. After confirming the absence of blood, cerebrospinal fluid, and gas, 5 mL of 0.5% ropivacaine was injected slowly. Successful blockade was indicated by the occurrence of Horner's syndrome postoperatively. Nerve blocks were administered on the contralateral side every other day, with a total of six treatments. Dynamic assessments were conducted before the treatment, after 1 week, after 2 weeks, and after 3 weeks. The patients' pruritus was evaluated using the 5-D Pruritus Scale, assessing itch duration, severity, direction of progression, incapacitation, and distribution, the results are shown in Table 1. There was further supplemented with the Peak Pruritus Numerical Rating Scale and the Twelve-Item Pruritus Scale, the results are presented in the Supplementary material (Elman et al., 2010; Jang et al., 2020). Additionally, the impact of the treatment on sleep quality was assessed using the Pittsburgh Sleep Quality Index (PSQI; Buysse et al., 1989), with results shown in the following tables and figures. Throughout the treatment and follow-up periods, we closely monitored the patients for adverse effects, such as localized pain and nerve injury. However, among the three participants in this study, only transient Horner's syndrome was observed following the nerve block, with no other adverse effects reported. These findings suggest that this therapeutic approach is associated with a high level of safety.

**Table 1:** 5-D pruritus scale.

**Case**	**Time**	**Duration**	**Degree**	**Direction**	**Disability**	**Distribution**	**Total score**
Case 1	Pre-treatment	4	5	5	5	4	23
	1 weeks later	2	2	2	2	4	12
	2 weeks later	2	2	3	2	2	11
	3 weeks later	2	3	3	2	2	12
Case 2	Pre-treatment	4	4	4	4	2	18
	1 week later	2	2	2	2	2	10
	2 weeks later	2	2	2	2	2	10
	3 weeks later	2	3	2	2	2	11
Case 3	Pre-treatment	4	5	4	5	4	20
	1 week later	2	3	2	2	2	11
	2 weeks later	2	3	3	2	2	12
	3 weeks later	2	3	2	2	2	11

During the follow-up period, the severity of the patient's symptoms and their impact on quality of life were assessed. The results of the study indicated that after six sessions of sympathetic nerve block treatment, all patients experienced a significant reduction in itch scores and an improvement in sleep quality. These findings suggest that sympathetic nerve block is clinically effective in improving symptoms of CKD-aP.

Sympathetic blockade has been demonstrated to be a safe and effective therapeutic approach, primarily through its mechanism of inhibiting the release of chemical signals associated with various disease states by blocking sympathetic nerve conduction (Baig et al., 2017; Lipov et al., 2017). By disrupting the conduction of sympathetic nerve signals, it effectively reduces the transmission of pain signals to the central nervous system, thereby alleviating pain perception. Moreover, by inhibiting the release of neurotransmitters such as norepinephrine from sympathetic nerve terminals, sympathetic blockade can further reduce pain perception, showing efficacy in conditions like neuropathic pain and cancer pain (Zhang et al., 2022; Lipov et al., 2009). Sympathetic blockade also exerts regulatory effects on the cardiovascular system, including decreasing heart rate, reducing myocardial contractility, and promoting vasodilation, which in turn reduces cardiac workload and alleviates symptoms associated with cardiovascular disorders (King et al., 2007; Wang et al., 2023). Additionally, sympathetic blockade can modulate immune system function by interrupting sympathetic innervation to immune organs, offering therapeutic potential in immune-related conditions such as ulcerative colitis (Lipov et al., 2020). It also has therapeutic applications in managing vasodilatory abnormalities associated with spasms, as well as in alleviating symptoms like hot flashes during menopause (Feigin et al., 2023). Sympathetic nerve block techniques are now widely used in clinical practice. With ultrasound-guided visualization, the safety of this procedure is significantly enhanced.

There is limited experimental evidence to demonstrate the effectiveness of sympathetic nerve block therapy in alleviating pruritus in patients with uremia. Our study may provide new insights into the potential clinical application of this therapy. We hypothesize that its mechanism of action may involve several factors. In patients with uremia, impaired renal function prevents the timely removal of metabolic waste products, leading to the accumulation of substances such as urea, creatinine, and uric acid in the bloodstream. This accumulation not only damages organs such as the liver and kidneys but may also affect both central and peripheral nervous systems, potentially stimulating cutaneous nerve endings and triggering pruritus (Wang et al., 2016). Sympathetic nerve block alleviates pruritus by interrupting sensory signal transmission in nerve endings. Furthermore, immune dysregulation plays a crucial role in CKD-aP, with levels of inflammatory mediators such as histamine, IL-2, and IL-6 being significantly elevated in patients with CKD-aP compared to healthy individuals (Ko et al., 2023). Immune organs are heavily innervated by sympathetic fibers, and sympathetic blockade modulates immune system activity by interrupting sympathetic innervation to these organs, thereby influencing the levels of both pro-inflammatory cytokines (e.g., IL-6, TNF-α) and anti-inflammatory cytokines (e.g., IL-10, TNF-β; Lipov et al., 2020). Immune cells express both α-adrenergic and β-adrenergic receptors, and the expression levels of these receptors vary depending on the cellular state, maturity, or activation status. Studies have demonstrated that adrenergic receptors functionally influence immune cell activities, particularly their migration, activation, and cytokine production (Schiller et al., 2021).

This case report has several limitations that warrant consideration. Notably, the absence of standardized quality-of-life assessment instruments, particularly the SF-36 or EQ-5D scales, may have compromised the objective quantification of the intervention's overall therapeutic efficacy. Future investigations should prioritize the implementation of large-scale, adequately powered, multicenter randomized controlled trials to establish comparative effectiveness between this therapeutic modality and conventional treatment paradigms. Furthermore, extending the follow-up duration to encompass several months or years would facilitate a comprehensive evaluation of the long-term clinical outcomes associated with sympathetic nerve blockade in the management of pruritus symptoms.

In conclusion, sympathetic nerve blockade represents a novel therapeutic strategy that offers a promising approach for the management of CKD-associated pruritus (CKD-ap). Although limited by a small sample size, our preliminary findings demonstrate clinically meaningful improvements in both pruritus severity and sleep quality among treated patients. However, additional experimental research and well-designed clinical trials are required to substantiate the safety and efficacy of this intervention before its widespread clinical application.

## Data Availability

The original contributions presented in the study are included in the article/Supplementary material, further inquiries can be directed to the corresponding author.
