# Supplementary material for: Case Report: Sympathetic nerve block treat chronic kidney disease-associated pruritus
Source: Front Neurosci. 2025 Apr 16;19:1529183. doi: 10.3389/fnins.2025.1529183 (PMC12040867; doi:10.3389/fnins.2025.1529183)
Supplement: Supplementary file 1 [file Data_Sheet_1.docx]

|  |  |  | 5-D Pruritus Scale | Peak Pruritus Numerical Rating Scale | Twelve-Item Pruritus Scale |
| --- | --- | --- | --- | --- | --- |
| Case 1 |  | pre-treatment | 23 | 9 | 17 |
|  |  | 1 week later | 12 | 5 | 16 |
|  |  | 2 week later | 11 | 6 | 16 |
|  |  | 3 week later | 12 | 6 | 15 |
| Case 2 |  | pre-treatment | 18 | 9 | 17 |
|  |  | 1 week later | 10 | 4 | 11 |
|  |  | 2week later | 10 | 5 | 9 |
|  |  | 3 week later | 11 | 5 | 12 |
| Case 3 |  | pre-treatment | 20 | 10 | 20 |
|  |  | 1 week later | 11 | 6 | 11 |
|  |  | 2 week later | 12 | 6 | 13 |
|  |  | 3 week later | 11 | 6 | 15 |

Supplementary table 1

Supplementary table 2

| PSQI score |  | pre-treatment | 1 week later | 2 week later | 3 week later |
| --- | --- | --- | --- | --- | --- |
|  | Case 1 | 10 | 5 | 5 | 5 |
|  | Case 2 | 12 | 6 | 6 | 7 |
|  | Case3 | 9 | 5 | 4 | 4 |

Supplementary figure 1

Supplementary figure 2

Supplementary figure 3
